# A mixed-methods evaluation of hepatitis B knowledge, attitudes, and practices among migrant women in Thailand

**DOI:** 10.1186/s12884-021-03914-2

**Published:** 2021-07-23

**Authors:** M. Bierhoff, A. H. Hashmi, C. Pateekhum, W. Jiraporncharoen, W. Wiwattanacharoen, MK Paw, F. H. Nosten, M. J. Rijken, M. Van Vugt, R. McGready, C. Angkurawaranon

**Affiliations:** 1 Shoklo Malaria Research Unit, Mahidol-Oxford Tropical Medicine Research Unit, Mahidol University, 63110 Mae Sot, Thailand; 2 Division of Infectious Diseases, Academic Medical Center, University of Amsterdam, Amsterdam, The Netherlands; 3 Department of Family Medicine, Faculty of Medicine, Chiang Mai University, 50200 Chiang Mai, Thailand; 4 Sarapee Hospital, 50140 Chiang Mai, Thailand; 5 Centre for Tropical Medicine and Global Health, Nuffield Department of Medicine Research Building, University of Oxford, Oxford, UK; 6 Department of Obstetrics and Gynaecology, Amsterdam UMC, University of Amsterdam, Amsterdam, the Netherlands; 7 Julius Global Health, The Julius Centre for Health Sciences, University Medical Centre Utrecht, Utrecht, Netherlands

**Keywords:** cross-sectional surveys, focus groups, Hepatitis B, interviews, observations, pregnancy, Mother-to-Child transmission, vaccination, qualitative research

## Abstract

**Background:**

Globally 90 % of transmission of Hepatitis B virus (HBV) is from mother-to child and occurs predominantly in resource limited countries where the prevalence of HBV is high. Transmission could be interrupted by timely vaccinations but coverage remains problematic in these areas. Low knowledge or awareness of HBV may play a part in low vaccination coverage. This study examines the provision of antenatal care counselling with a focus on HBV in two different regions of northern Thailand, Sarapee Hospital (SH), Chiang Mai, and Shoklo Malaria Research Unit (SMRU), Tak Province.

**Methods:**

A mixed-methods sequential explanatory study design was used to evaluate antenatal services for migrants. Cross-sectional knowledge, attitude and practice (KAP) surveys were conducted immediately after counselling at first ANC contact, at 3–6 months after first ANC contact and at delivery. Surveys provided quantitative data, and qualitative methods included observations, focus group discussions (FGD) and in-depth interviews (IDI); analysed thematically to explore concepts of knowledge and understanding, attitude and practice of pregnant women and providers.

**Results:**

Between September-2019 and May-2020, 757 women participated to KAP surveys, and 31 observations of counselling, 16 FGD and 9 IDI were conducted. KAP surveys showed in spite of low knowledge about HBV transmission, infection, or vaccination (correct response: SH 5.7 %, 9/157; SMRU 34.0 %, 204/600), most women (≥ 93 %, either site) understood they were screened for HBV and were willing to vaccinate infants for HBV. In explaining KAP survey results, qualitative analysis suggests counselling should: use the appropriate language; be tailored to the local health literacy level, provide only pertinent information, be repeated over the antenatal period; and attempt to ensure patient privacy (where possible). Programme effectiveness benefits from positive attitudes to screening and vaccinations and a high level of trust in the providers nevertheless participants provided good suggestions for improvements of the service.

**Conclusions:**

Limited knowledge of HBV among migrant women can be improved by counselling that emphasizes actionable knowledge such as vaccination schedule. Key improvements to the counselling process include training counsellors to conduct interactive counselling sessions in the woman’s language, using appropriate visual aids and timely repetition over the course of the antenatal period.

## Background

Globally, mother-to-child transmission (MTCT) of hepatitis B virus (HBV) accounts for more than 90 % of all acquired HBV infections [1]. Congenitally acquired HBV is associated with the highest risk of chronic HBV infection that can lead to cirrhosis, hepatocellular carcinoma and death [2]. Completion of at least three doses of HBV vaccine, with the first given within 24 h of birth, and administering hepatitis B immunoglobulin (HBIG) when needed, remains challenging [3]. Although global coverage for the three HBV vaccines as part of Expanded Programme on Immunization (EPI) is high at around 85 % [4, 5], disparities exist such as in Southeast Asia where coverage for the birth dose remains much lower at 54 % for the South East Asia region. These disparities can be sizeable even between bordering southeast Asian nations; in 2019, the birth dose had 99 % coverage in Thailand compared to only 17 % in Myanmar [6–8].

Although ensuring high HBV vaccine coverage is multifactorial, at an individual level, improving awareness of HBV and rates of screening for HBV during pregnancy can prevent MTCT. Low awareness of HBV was almost universally reported from cross-sectional surveys of pregnant women published in the past 5 years from North America [9], Africa [10–17], Asia [18–21], Eurasia [22], the middle East and the Pacific [23, 24]. In South East Asia, 10.8 % of pregnant Vietnamese women correctly answered questions about HBV directly post counselling [25], while in Laos only 24.5 % were aware of the HBV vaccine [21].

In a 2015 study, migrant women in Chiang Mai and Tak Provinces in Northern Thailand, had a near universal uptake of HBV point-of-care-testing, ≥ 93 % infants received the birth dose but less than half of the infants completed three EPI HBV vaccinations at the hospital where they delivered [26]. Antenatal care (ANC) provides an opportunity to communicate with and support women, families and communities at a critical period of the life-course, [27–30] but few studies have assessed if ANC counselling improves knowledge or HBV vaccine coverage rates [31]. This study takes a mixed-methods approach to examine the counselling process as it relates to knowledge, attitude and practice (KAP) of migrant pregnant women, and explore factors related to migrant women’s understanding of HBV across two different provinces of Thailand.

## Methods

### Setting

A 2017 UN migration report estimated Thailand to have over 3 million international migrants and Tak Province, Thailand, to have up to 200,000 non-Thai migrants from Myanmar. Migrant ANC is provided by the informal sector including non-governmental clinics for over three decades [32, 33], in addition to the formal, public Thai government facilities. This study was designed in parallel, across two ANC programmes offering care for migrant pregnant women: (1) Sarapee Hospital (SH), Chiang Mai Province, where migrant ANC is included within the broader Maternal and Child Health services, and is principally provided by Thai government facilities; and (2) in non-government services along the Thailand-Myanmar border from Shoklo Malaria Research Unit (SMRU) [34, 35]. Two SMRU clinics participated, one approximately 30 km north and one approximately 60 km south, of the town of Mae Sot, Thailand. At SMRU the women generally speak Burman or Karen with two dialects for Karen language, Sgaw and Poe.

### Counselling services for pregnant women

Pre-test counselling provided information on a number of pregnancy-related topics including HBV and was routine at all sites for women at the first ANC contact. Post-test counselling at SH was provided to women returning for an ANC contact following the HBV screening whereas it was provided on the same day as the screening at SMRU. Pregnant mothers need to actively opt-out of the screening if they do not want testing. No further counselling about HBV is routinely provided after the first ANC visit. The study focuses on HBV given that rates of HBV in migrant women are 3.6 to 23 times higher than HIV at first ANC screening in pregnant women at SH and SMRU, respectively [26].

### Study design

**Fig. 1:**
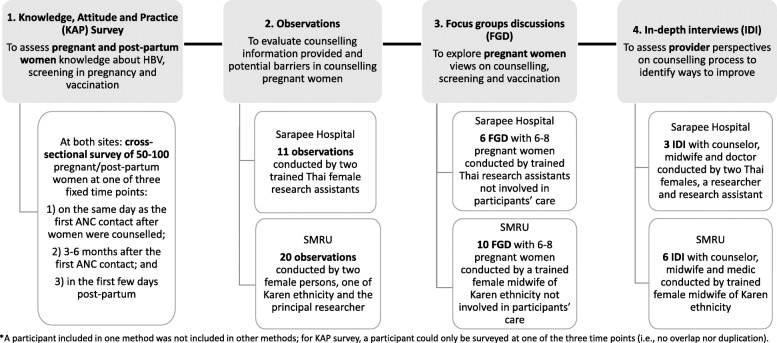
Summary of mixed-methods used in the study in the order they were carried out at two sites: Sarapee Hospital, Chiang Mai, Thailand and Shoklo Malaria Research Unit (SMRU) migrant clinics, Tak Province, Thailand

A mixed-methods sequential explanatory study design was used to evaluate ANC counselling and services for migrants with a focus on HBV. Knowledge, attitude and practice (KAP) survey provided quantitative data. Qualitative methods included observations, focus group discussions (FGD) and in-depth interviews (IDI). The techniques were sequential and iterative with the qualitative methods following the KAP survey, while maintaining a focus on three major concepts: understanding, attitude, and practices (Fig. 1). If a participant was included in one method, they were not included in other methods to avoid duplication and contamination.

### KAP survey

The KAP survey instrument was developed from published surveys to assess sociodemographic characteristics of study participants, participant knowledge about HBV screening and management in pregnancy and children, as well as attitudes toward HBV screening [20]. Cross-sectional surveys were conducted at three different time points during pregnancy with 50–100 participants each time, at each site, with participants included only once. Survey time points were: (1) while awaiting test results on the same day as the first ANC contact after women were provided pre-test counselling (as described above); (2) three to six months after the first ANC contact; and (3) in the first few days post-partum. All women that attended the clinics during the study period were approached for participation in the study and if informed consent was forthcoming they completed the KAP survey.

### Qualitative methods: observations, focus group discussions, in-depth interviews

At each site, the research team observed at least ten counselling sessions. These sessions could be pre-test or post-test. An observation form helped guide observations. Observers were trained to observe communication and languages used, the time taken to counsel as well as the amount of time for each topic, and to make general observations of the environment.

FGD involved 6–8 pregnant women that were purposively sampled to create groups similar in ethnicity and language to create the most comfortable setting for free and open discussion. Participants interested in participating in the FGD were provided with information regarding the objectives of the FGD. FGD included questions specifically designed to probe pregnant women about their views on counselling, screening and vaccination. The FGD were conducted in separate rooms that promoted participant privacy.

Nine IDI were conducted, three per site, with service providers including counsellors, midwives and doctors or medics. IDI took place to explore what they thought could improve or what they would change to support counselling and ANC services. The IDI took place at a site and time of the interviewee convenience.

The qualitative methods used and their main objectives were summarized (Fig. 1). Observations at SMRU and at SH were conducted by two female persons, all with experience of providing care in mother and child health but not currently providing care at the sites. The FGD and IDI at SMRU were conducted by a trained female midwife (Karen), fluent in the preferred language of the participants with no prior relationship with the women and who was not responsible for their care. At SH the FGD were conducted in Thai by trained research assistants (Thai) not involved in service provision at SH. At SH the IDI were conducted by two women working in the Family Medicine Department at SH. Additional facilitators were at times included in FGD/IDI; research team members involved with FGD and IDI at either site had no prior relationships with pregnant women nor were they directly responsible for participants’ care.

### Analysis

Quantitative data was analysed using SPSS version 23 (SPSS Inc., Chicago IL, USA). “All Knowledge answers correct” resulted from correct answers to each of the seven knowledge questions about transmission, HBV infection and vaccination.

IDI and FGD were audio recorded and the audio files were transcribed and translated into English by an independent translator. NVivo 12 (QSR International, Melbourne, Australia) was used for analysing qualitative data. Qualitative data was analysed using a conceptual framework. We used a constructive approach, which assumes reality is determined by the context and perspective of the participants, and that multiple truths are possible. Data-driven inductive thematic analysis was used to scrutinize the data to identify and analyse patterns across the dataset using constant comparison techniques. For each site, investigators performed line-by-line coding according to *a priori* codes based on the existing literature and tailored to the local study context [14, 36, 37]. Once definitions of codes were agreed upon between investigators, coded transcripts were compared between the investigators. Discrepancies were discussed and codebooks were revised accordingly. The final codebook was agreed upon and transcripts re-coded subsequently. Inductive thematic analysis was performed, discussed by the investigators and confirmed by the rest of the research team [38].

## Results

Between September 2019 and May 2020, 789 KAP surveys, 31 observations of ANC counselling, 16 FGD and 9 IDI were conducted (Fig. 1).

### Knowledge, attitudes and practices of pregnant women

The KAP survey was completed by 757 women (251 at first ANC, 253 at 3–6 months after first ANC and 253 at delivery, Table 1); none of whom opted out of screening. The majority of respondents were of Myanmar nationality, 97.5 % (769/789). Of the respondents from SH, 89/157 (57 %) received no prior education compared to 144/600 (24 %) in SMRU. Respondents that did receive education, the median level ranged from 2 to 7 years at SMRU and 4–12 years at SH.

**Table 1:** Demographics of the women participating to the KAP survey

Variable		First ANC (*n* = 251)		3–6 months after first ANC (*n* = 253)		Delivery (*n* = 253)	
		Sarapee Hospital (*n* = 51)	SMRU (*n* = 200)	Sarapee Hospital (*n* = 53)	SMRU (*n* = 200)	Sarapee Hospital (*n* = 53)	SMRU (*n* = 200)
Age, median [IQR] (min-max)		26 [22-29] (16–41)	25 [21-30] (15–45)	27 [23-30] (16–39)	25 [20-30] (14–46)	25 [22-32] (17–47)	24 [20-29] (16–44)
Nationality n(%)	Thai-Hilltribe	5 (9.8)	0	3 (5.8)	0	1 (1.9)	0
	Myanmar	46 (90.2)*	200 (100.0)	49 (94.2)	200 (100.0)	50 (96.1)	200 (100.0)
Insurance n(%)	Yes	44 (86.3)	83 (41.5)	39 (73.6)	200 (100.0)	46 (88.5)	186 (93.0)
Ethnicity n(%)	Thai	0	0	0	0	0	1 (0.2)
	Burman	1 (2.0)	100 (50.0)	2 (3.8)	91 (45.5)	1 (1.9)	92 (47.2)
	Shan/Thai Yai	43 (84.3)	4 (2.0)	43 (81.1)	1 (0.5)	47 (88.7)	11 (2.7)
	Karen	0	92 (46.0)	0	97 (48.5)	0	90 (46.5)
	Other	7 (13.7)	4 (2.0)	8 (15.1)	11 (5.5)	5 (9.4)	6 (3.3)
Years of stay current residence, median [IQR] (min-max)		8 [4-15] (1–25)	3 [< 1–10] (< 1 to 30)	6 [4-12] (1–30)	5[1-10] (< 1 to 30)	8 [4-16] (1–27)	5 [1-13] (< 1 to 32)
No children yet, n% (parity = 0)		21 (41.0)	78 (39.0)	29 (55.0)	92 (46.0)	0	0
Number of children (if not ‘no’), median [IQR] (min-max)		1 [1-2] (1–3)	2 [1-2] (1–6)	1 [1-1] (1–2)	2 [2-3] (1–6)	2 [1-1] (1–2)	2 [1-3] (1–6)
No education n(%)		27 (54.0)	50 (25.0)	30 (56.6)	54 (27.0)	32 (61.5)	40 (24.0)
If education, highest education grade, median [IQR] (min-max)		6 [4-10] (1->12)	4 [2-7] (1–12)	9 [5-12] (2–12)	4 [3-7] (1–11)	7 [4-9] (1->12)	4 [3-7] (1–10)
Proportion HBsAg positive# n(%)		1 (2.0)	5 (2.5)	2 (3.8)	2 (1.0)	4 (7.5)	10 (5.0)

*Includes one woman who self-identified as Cambodian; # HBsAg positive at the different survey time points (First ANC, 3–6 months after first ANC, Delivery) not tested for Hepatitis B at this time point [as all women were tested at their first ANC visit]

Abbreviations: ANC antenatal care; SMRU, Shoklo Malaria Research Unit migrant clinics

### Knowledge

**Fig. 2:**
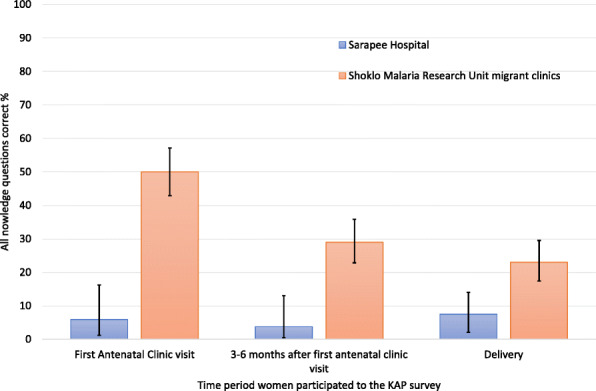
Percentage of respondents answering all knowledge questions correctly at different time points in pregnancy at each site

Knowledge about HBV was low according to the KAP surveys conducted directly after the first ANC counselling: only 5.9 % (3/51) at SH and 50.0 % (100/201) at SMRU answered all questions about HBV correctly (Table 2). Although HBV knowledge at SH was low at every survey point in pregnancy, there was a significant decline in knowledge among women from SMRU compared to baseline, at 3–6 months after the first ANC 29.0 % (58/200) and at delivery 23.0 % (46/150) (*p* < 0.001, Fig. 2), but not between the two later time-points. Over 57 % (89/157) of women from SH correctly answered that HBV is transmissible from mother-to-child but less than one in five women, 18.6 % (29/157), were aware of the existence of a HBV vaccine. In contrast, women at SMRU knew about HBV transmission, especially from mother-to-child (93.2 %, 559/600), and nearly three in four women (72.5 %, 435/600) were aware of a HBV vaccine.

**Table 2:** Women’s response to knowledge component of KAP survey questions (n, %)

Items	Response	Total (*n* = 757)		First ANC (*n* = 251)		3–6 months after first ANC (*n* = 253)		Delivery (*n* = 253)	
		Sarapee Hospital (*n* = 157)	SMRU (*n* = 600)	Sarapee Hospital (*n* = 51)	SMRU (*n* = 200)	Sarapee Hospital (*n* = 53)	SMRU (*n* = 200)	Sarapee Hospital (*n* = 53)	SMRU (*n *= 200)
**All Knowledge answers correct**		9 (5.7)	204 (34.0)	3 (5.9)	100 (50.0)	2 (3.8)	58 (29.0)	4 (7.5)	46 (23.0)
HBV can be transmitted through blood transfusion	Yes	68 (43.3)	497 (82.8)	25 (49.0)	177 (88.5)	18 (34.0)	170 (85.0)	25 (47.2)	150 (75.0)
	No	2 (1.3)	39 (6.5)	0	13 (6.5)	0	10 (5.0)	2 (3.8)	16 (8.0)
	Don’t know	87 (55.4)	64 (10.7)	26 (51.0)	10 (5.0)	35 (66.0)	20 (10.0)	26 (49.1)	34 (17.0)
HBV can be transmitted through unprotected sexual intercourse	Yes	62 (39.5)	485 (80.8)	17 (33.3)	179 (89.5)	22 (41.5)	155 (77.5)	23 (43.3)	151 (75.5)
	No	7 (4.5)	52 (8.7)	4 (7.8)	15 (7.5)	1 (1.9)	18 (9.0)	2 (3.8)	19 (9.5)
	Don’t know	88 (56.1)	63 (10.5)	30 (58.8)	6 (3.0)	30 (56.6)	27 (13.5)	28 (52.8)	30 (15.0)
HBV can be transmitted from mother to fetus	Yes	89 (57.1)	559 (93.2)	27 (52.9)	195 (97.5	30 (57.7)	186 (93.0)	32 (60.4)	178 (89.0)
	No	2 (1.3)	12 (2.0)	0	3 (1.5)	1 (1.9)	4 (2.0)	1 (1.9)	5 (2.5)
	Don’t know	65 (41.7)	29 94.8)	24 (47.1)	2 (1.0)	21 (40.4)	10 (5.0)	20 (37.7)	17 (8.5)
HBV can be transmitted through the unsafe use of needles and sharps	Yes	91 (58.3)	532 (88.7)	32 (62.7)	183 (91.5)	27 (51.9)	182 (91.0)	32 (60.4)	167 (83.5)
	No	3 (1.9)	31 (5.2)	1 (2.0)	13 (6.5)	0	9 (4.5)	2 (3.8)	9 (4.5)
	Don’t know	62 (39.7)	37 (6.2)	18 (35.3)	4 (20)	25 (48.1)	9 (4.5)	19 (35.8)	24 (12.0)
HBV can cause damage to the liver	Yes	83 (53.2)	488 (81.3)	28 (54.9)	183 (91.5)	23 (44.2)	164 (82.0)	32 (60.4)	141 (70.5)
	No	2 (1.3)	18 (3.0)	0	5 (2.5)	1 (1.9)	3 (1.5)	1 (1.9)	10 (5.0)
	Don’t know	71 (45.5)	94 (15.7)	23 (45.1)	12 (6.0)	28 (53.8)	33 (16.5)	20 (37.7)	49 (24.5)
A person can be infected with HBV and not show any signs	Yes	53 (34.0)	377 (62.8)	15 (29.4)	149 (74.5)	21 (40.4)	116 (58.0)	17 (32.1)	112 (56.0)
	No	9 (5.8)	146 (24.3)	2 (3.9)	47 (23.5)	4 (7.7)	56 (28.0)	3 (5.7)	43 (21.5)
	Don’t know	94 (60.3)	77 (12.8)	34 (66.7)	4 (2.0)	27 (51.9)	28 (14.0)	33 (62.3)	45 (22.5)
There is a vaccine for HBV	Yes	29 (18.6)	435 (72.5)	10 (19.6)	167 (83.5)	8 (15.4)	135 (67.5)	11 (20.8)	133 (66.5)
	No	13 (8.3)	67 (11.2)	3 (5.9)	24 (12.0)	3 (5.8)	23 (11.5)	7 (13.2)	20 (10.0)
	Don’t know	114 (73.1)	98 (16.3)	38 (74.5)	9 (4.5)	41 (78.8)	42 (21.0)	35 (66.0)	47 (23.5)

Abbreviations: ANC antenatal care; HBV, Hepatitis B virus; SMRU, Shoklo Malaria Research Unit migrant clinics

### Attitudes and practice

Despite limited knowledge about HBV, most women knew that they were getting tested for the disease (725/757 (95.8 %) and nearly all women wanted to get their infant vaccinated (741/757 (97.9 %), Table 3). A variable proportion (54–98 %) of women informed their husbands, more at SH than at SMRU, of their status but the reasons for this were not further explored (Table 3).

**Table 3:** Women’s response to attitude and practice components of KAP survey questions (n, %)

Items	Response	Total (*n* = 757)		First ANC (*n* = 251)		3–6 months after first ANC (*n* = 253)		Delivery (*n* = 253)	
		Sarapee Hospital (157)	SMRU (600)	Sarapee Hospital (51)	SMRU (200)	Sarapee Hospital (53)	SMRU (200)	Sarapee Hospital (53)	SMRU (200)
Did you know you were getting screened for HBV?	Yes	145 (93.5)	580 (96.7)	41 (80.4)	199 (99.5)	52 (100.0)	187 (93.5)	52 (100.0)	194 (97.0)
	No	10 (6.5)	20 (3.3)	10 (19.6)	1 (0.5)	0	13 (6.5)	0	6 (3.0)
Are you willing to vaccinate your baby for HBV?	Yes	153 (98.1)	588 (98.0)	50 (98.0)	199 (99.5)	50 (96.2)	194 (97.0)	53 (100.0)	195 (97.5)
	No	1 (0.6)	5 (0.8)	0	1 (0.5)	1 (1.9)	3 (1.5)	0	1 (0.5)
	Don’t know	2 (1.3)	7 (1.2)	1 (2.0)	0	1 (1.9)	3 (1.5)	0	4 (2.0)
Did you tell your spouse about your HBV status?	Yes	147 (94.2)	425 (70.8)	50 (98.0)	108 (54.0)	48 (92.3)	150 (75.0)	49 (92.5)	167 (83.5)
	No	7 (4.5)	174 (29.0)	0	92 (46.0)	3 (5.8)	50 (25.0)	4 (7.5)	32 (16.0)
	Don’t know	2 (1.3)	1 (0.2)	1 (2.0)	0	1 (1.9)	0	0	1 (0.5)

Abbreviations: HBV, Hepatitis B virus; SMRU, Shoklo Malaria Research Unit

### Counselling process

Out of the 31 observations performed, 16 were prior to HBV screening (pre-test) and 15 were after HBV screening (post-test). On average, ANC counselling took between 3 and 58 min, across all sites. At SH, six observations of pretest counselling were performed that lasted a median of 14.0 min (Inter quartile range (IQR) 8.5–15.0 min), compared to 28.5 min at SMRU (IQR 20.8–42.8) for 10 observations. Within the pre-test counselling period only a short window was dedicated to HBV: two minutes and three minutes for SH and SMRU, respectively. For the 15 post-test counselling sessions observed (SH 5 and SMRU 10), the median duration was 9.5 min (IQR 6–22), with HBV being discussed only once with one patient who tested HBsAg positive and received an additional 20 min counselling about HBV at SMRU.

The topics covered in the observed pre-test counselling that were common to both migrant settings included HIV, sexually transmitted infections and condom information, HBV, family planning, hygiene, infant EPI, anaemia, nutrition, smoking, congenital abnormalities and health insurance. SH additionally covered thalassaemia and alcohol, while SMRU additionally covered malaria, G6PD deficiency and neonatal jaundice.

At SH the counselling was performed one-on-one with the woman (and husband if present) in Thai language irrespective of the woman’s ethnicity or preferred language. There were no visual aids used for the counselling sessions, all of which—pre- or post-test counselling—were performed in an open clinic room with other people in the same space. At SMRU, pregnant women received pre-test counselling in a group, and while pre-test counselling was performed in a room separated from the rest of the antenatal care area, it did not have a door. SMRU staff would counsel using the local languages, but occasionally the groups were of mixed ethnicity and it was therefore possible that some women received counselling in something other than their native language. SMRU staff also used visual aids such as pictures and a condom demonstration. SMRU staff conducted post-test counselling one-on-one with the patient in either the same area or a small private room.

### Qualitative exploration of factors related to knowledge and understanding, attitudes and practices

Observations, FGD and IDI, helped understand factors influencing knowledge, attitude, and practices determined by KAP surveys. For factors that influence knowledge and understanding, two main themes were identified: (1) the content of ANC counselling and (2) language and cultural considerations. For factors that influence the attitudes and practices of migrant women, two main themes were identified: (1) attitudes towards screening and vaccines; and (2) trust in provider.

#### Factors that influence knowledge and understanding

*1) the content of ANC counselling*.

The observations indicated that a multitude of topics were covered in a short amount of time—a finding confirmed by both FGD and IDI. Many women in FGD reported they had difficulty understanding and remembering the ANC counselling provided given the large breadth of topics covered.

***“… there was a lot of information so I can’t remember and I mixed them up.” [Pregnant woman, FGD, SMRU]***.

***“… I didn’t pay attention, there was too much information.” [Pregnant woman, FGD, SH]***.

One SMRU staff explained that there are many topics that are explained in a short amount of time which can be a cause for confusion among women, and some women in the FGD realized counselling was not repeated, implying that it might be helpful.

***“It’s been quite long so I can’t remember, the nurse only taught me once, I need to learn multiple times to understand. I sometimes understand and sometimes don’t, since the language is hard and I don’t know Thai that much.” [Pregnant woman, FGD, SH]***.

In addition to the manner in which the counselling was delivered, it is likely that the content was not appropriate to the population receiving it. This may have been more so the case in SH where we note a larger proportion of women without any formal education (Table 1). There were misconceptions about the transmission route (food related, alcohol, smoking) and there was a clear mix up with neonatal jaundice at SMRU. These may also partly explain the findings of low knowledge from the KAP survey.

***“One infant near my home suffered from it (ref HBV) before. The baby was brought to this clinic and also sunbathed at home.” [Pregnant woman, FGD, SMRU***].

Health literacy was a noted concern among health workers.

***“Most women who do not read could not understand. They cannot answer when they are asked. They are told about 3 different diseases so they mix up the information and can’t remember all the information.” [Counsellor, IDI, SMRU]***.

To help convey health information where health literacy may be limited, SMRU—as in many low resource settings—employs visual aids. Many participants in IDI suggested that visual aids, especially media with many pictures, could improve understanding when health literacy and language were considerable barriers. Although visual aids could be useful, observations suggested that at SMRU visual aids may not have been presented to women well at times, and that some visuals may have been misinterpreted by health workers themselves. Visual aids should be reviewed for patient understanding. Health workers should receive continuing education/refresher training for health workers, as counsellors themselves acknowledged their own lack of training and knowledge as a barrier to effective counselling.

***“I want to increase the nurse’s potential, their confidence in counselling in terms of knowledge and communication method to help patients understand.” [Doctor, IDI, SH]***.

*2) Language and cultural considerations*.

Language was a key issue throughout this study, likely resulting in the difference seen in the knowledge portion of the KAP surveys between SH and SMRU. In both sites misconceptions about HBV were common, although of a slightly different nature. At SMRU misconceptions were mostly a mix up of HBV with diseases like neonatal jaundice. At both SH and SMRU, there were misconceptions about transmission such as beliefs that HBV could be transmitted through food.

***“I’m afraid because this disease could come from vegetables and chemicals in meat.” [Pregnant woman, FGD, SH]***.

***“The disease can be transmitted to you if you use the same soap to take a shower as other people and when you stay close to someone and they sneeze.” [Pregnant woman, FGD, SMRU]***.

Observations, FGD, and IDI demonstrated the manner in which language was important, specifically the diversity of languages spoken in the study population and the language used to communicate between the counsellor and the woman. Table 1 shows this diversity, with Shan ethnicity the most common among migrants in SH, but with nearly 15 % of the population claiming a distinct ethnicity (“other”), predominantly from Myanmar, and speaking Burmese, Shan, or other local languages. Regardless of the local language spoken by patients at SH, all counselling was performed in Thai. In contrast, at SMRU, two ethnic groups predominate (Burman and Karen), but a variety of languages are spoken such that counselling is often performed in Burmese in groups of mixed ethnicity. This diversity is apparent as it was inadvertently replicated as part of this study, as research teams were unable to completely separate FGD according to ethnicity and language, with half of all FGD across both sites of mixed ethnicity/preferred language. The language barrier was highlighted as one of the biggest issues in terms of the ability to understand the counselling information.

***“I sometimes understand and sometimes don’t, since the language is hard and I don’t know Thai that much.” [Pregnant woman, FGD, SH]***.

***“I didn’t understand the language so I didn’t understand what the doctor taught.” [Pregnant woman, FGD, SH]***.

Health workers noted that using appropriate language during counselling could improve understanding.

***“We have to ask them clearly what language they are best at and to give the information in the language they understand best.” [Medic***, ***IDI, SMRU]***.

The qualitative methods help to illustrate the nature of counselling in these ANC settings and provide cultural and practical considerations that can be taken into account. Observations show that privacy at either site was difficult to ensure with other clinic attendees able to see and hear women. The FGD and IDI highlight practical considerations that can make learning information difficult due to distractions such as children at play or health workers coming in and out of the counselling rooms, or external noises from generators or fans. Cultural considerations could also make for a difficult “learning” environment: in FGD, we asked women if they had difficulty understanding, why they didn’t ask the health workers for clarification. As one woman explained,

***“I didn’t ask before because there were many people and I didn’t want to interrupt the doctor. If there was an opportunity to ask, I would.” [Pregnant woman, FGD, SH]***.

IDI participants also had mentioned the lack of interaction during counselling between patient and provider. Often if patients were silent, health workers understood this to mean that they had no additional questions.

***“This (referring to no questions asked) makes us think that they understand, but they don’t. And they obey us, coming for antenatal care. They just do everything we say***.**”*[nurse, IDI, SH]***.

Although health workers focused on language as the significant barrier, FGD participants often provided more nuance suggesting cultural, as opposed to linguistic, concerns. Participants may have not wanted “to interrupt the doctor”, to be burdensome to health care staff, and or to seem as though they didn’t understand.

#### Factors that influence attitudes and practices

1) *attitudes towards screening and vaccinations*

Even though KAP surveys, observations, FGD, and IDI suggest much confusion about the details of HBV, women still grasped the importance of being screened for HBV and infant vaccinations. In all FGD, women highlighted the importance of screening for diseases in pregnancy, and specifically for HBV. As women learned more about HBV, understanding the means of preventing HBV assuaged their initial concern for the infection.

***“ If I have the disease I have to protect others from it.” [Pregnant woman, FGD, SH]***.

***“I will learn more about the disease and ways of protection. Then I’ll teach my kid to be careful not to become infected.” [Pregnant woman, FGD, SH]***.

Women expressed their willingness to share information about HBV, including their own status, with other people without hesitation and this sentiment was found across all sites.

***“I thought about telling my results to others so maybe others can help, not worried or anything.” [Pregnant woman, FGD, SH]***.

***“I can also share the information with people around me.” [Pregnant woman, FGD, SMRU]***.

2) *Trust in health services provided*

During the FGD, women expressed wanting to do what health care workers advised, although the pathophysiological aspects of HBV and its transmission may have been lost on them. This, taken together with the women’s positive attitudes towards being screened and having their infants vaccinated, suggested trust in the health system, and a strong belief that health care providers had the women’s best interests in mind.

***“If I have the diseases, then the health workers will give me medical treatment and can protect the child from getting the diseases.” [Pregnant woman, FGD, SMRU].***

***“I will ask the nurses for advice and follow their advice.” [Pregnant woman, FGD, SH]***.

This trust likely explains why women didn’t feel coerced to undergo HBV testing. When asked, women agree that testing the blood for diseases is important and they generally want to know the results.

***“The health workers told me about what diseases I will be tested for and ask my permission before doing the test for me.” [Pregnant woman, FGD, SMRU]***.

***“The health workers told me that my blood will be drawn. I think it’s good for my child and myself. I will not know if I have any disease or not if my blood was not drawn.” [Pregnant woman, FGD, SMRU]***.

## Discussion

This study provided an in-depth evaluation of antenatal counselling about HBV for migrant women in a rural and urban setting in Southeast Asia. A former study in the same population of migrants identified < 50 % of infants completed the HBV vaccinations at the hospital of birth [26]. This study identified a number of factors that may help improve the delivery of knowledge and understanding of HBV for the migrant population, and in turn, improve overall utilization of HBV prevention measures in these communities.

### Problems with current ANC HBV counselling

The KAP survey highlighted that immediately after receiving HBV counselling, women’s knowledge of HBV was low and this is consistent with previous publications globally [9–25]. Given that women did not receive additional HBV counseling after the first ANC visit, we expected a decline in knowledge for women tested at delivery—suggesting that remembering the key messages was challenging. Accessing good counselling about HBV during ANC is difficult due to barriers with regard to content, language and cultural sensitivity, and not tailoring counselling to the health literacy of the population. In this setting, although the proportion of women with HBV significantly outweighs the proportion with HIV, observations of counselling sessions seemingly ignored this fact, placing greater emphasis on HIV compared to HBV information. Improvements may include: interactive sessions that provide more pertinent, actionable information from the woman’s perspective; allowing adequate time for each session; repeating these key messages about infant HBV vaccinations [9] over the course of ANC; and identifying spaces for counselling that ensure privacy.

### The effect of knowledge on practice

Evidence suggests knowledge can have a tremendous impact on practices [39] but paradoxically, low knowledge of HBV measured by the KAP in migrant women did not impact practice in terms of uptake of screening and willingness to vaccinate their infants. At SH almost all of the women were willing to give their baby the appropriate vaccinations but very few knew that there was a vaccine for HBV. This indicates that the counselling about HBV should focus on providing clear information for when, where and how often women should get vaccines for their children, instead of the disease itself. This does not mean that information about HBV should be withheld during counselling, but the focus should be that the woman understands the important role of vaccination in preventing infection in her infant. After initial screening information about HBV during the first ANC visit, follow up visits should repeat the vaccination message, not merely for HBV but also for other communicable diseases like tetanus.

### Limitations

Limitations include the language of the KAP and FGD: at SMRU Sgaw and Pwo Karen dialects were not done separately, and at SH only Thai language was used, which likely contributed to low knowledge scores. The KAP was not done prior to ANC counselling so the baseline knowledge was not known and the same women was not followed across pregnancy for KAP survey which would be a more reliable measure of knowledge retention. Finally, the KAP tool itself could have been better tailored to the important questions e.g. “is there a HBV vaccine?”, which tests actionable knowledge. This is relevant to the global literature on HBV where vaccination is sub-optimal as KAP surveys may not accurately capture salient HBV knowledge in settings where health literacy remains a significant barrier.

### Next steps

In spite of the limitations, the next steps should be to improve the counselling process, determine the relevant information appropriate to this population and their risks in pregnancy, develop informative counselling materials, and ultimately ensuring documentation of vaccination status of children.

The process of counselling can be improved by focusing on different steps of HBV at different contact visits. During the first ANC visit attention should be given to HBV screening. Initiating the counselling about vaccinations later during ANC and at pre-discharge after birth might improve completion of infant vaccinations and reduce the volume of information at first ANC.

In the IDI it was mentioned that women might feel more supported if there was somebody else with them to hear the important information. The father of the child or a relative (such as the pregnant woman’s mother) would be obvious choices to be present during this post-delivery counselling. At the time of counselling most women in this analyses were without the support of their husbands and not all women disclosed results to their husbands. Community-based interventions that involve men might improve the overall awareness of HBV and preventive measures [40]. FGD drew connections to social barriers during counselling such as the perceived threshold for bothering the counsellor with their questions. If she feels supported by other people to ask questions this might resolve the barrier and thus improve the outcome of the counselling.

Materials that are being used to deliver the counselling should be improved to ensure correct information and enhance patient understanding. The language barrier is challenging in this population where multiple languages are spoken and it would be unattainable to have a licensed nurse present in the clinic that speaks all of these languages. A possible solution to overcome this barrier would be to use tested visual aids [41]. These visual aids could be pictures, graphics, and short messages that are tailored to the understanding of the target population. Other possibilities are digital and multi-media interactive technologies if this is possible in the clinic or if the woman has a smartphone. Previous studies on the usage of visual aids to help counsel pregnant women has shown great benefit but attention should be given to ensure the correct interpretation of the visual aid [42, 43].

Refresher training about HBV for counsellors would be important, especially to understand the burden of HBV in their communities as well as counselling skills. One of the methods that has a reported improved outcome in disease-specific knowledge, adherence and self-efficacy is the “teach back method” that was introduced in an attempt to reinforce education to patients [44]. To ensure that women understand the counselling and the preventive options available to them and are able to exercise an informed choice, having clinical guidelines that precisely inform how healthcare workers can facilitate informed screening choices without compromising patient autonomy would be beneficial [45].

## Conclusions

Migrant women, mostly from Myanmar, express trust in healthcare services in Thailand. Overall, limited knowledge of HBV among migrant women can be improved by counselling that emphasizes actionable knowledge such as vaccination schedule. Key improvements to the counselling process include training counsellors to conduct interactive counselling sessions in the woman’s language, using appropriate visual aids and timely repetition over the course of the antenatal period.

## Data Availability

Data cannot be shared publicly because of ethical requirements from Chiang Mai University. Data are available from the Faculty of Medicine, Chiang Mai University Ethics Committee (contact via researchmed@cmu.ac.th) for researchers who meet the criteria for access to confidential data.

## Abbreviations

ANC: Antenatal care
EPI: Expanded Programme on Immunization
FGD: Focus group discussions
HBIG: Hepatitis B immunoglobulin
HBV: Hepatitis B virus
IDI: Indepth interviews
IQR: Interquartile range
KAP: Knowledge, attitude and practice
MTCT: Mother-to-child transmission
SH: Sarapee Hospital
SMRU: Shoklo Malaria Research Unit
